# Obituary: Dr. Marie-José Walenkamp (1966-2021)

**DOI:** 10.3389/fendo.2021.806985

**Published:** 2021-12-09

**Authors:** Jan M. Wit

**Affiliations:** Willem-Alexander Children’s Hospital, Division of Pediatric Endocrinology, Leiden University Medical Center, Leiden, Netherlands

**Keywords:** growth, growth hormone, IGF-I - insulinlike growth factor 1, short stature, genetic defects

To the great sadness of her husband, daughters, parents, other family members, friends and colleagues, our long-time collaborator Dr. M.J.E (Marie-José) Walenkamp, pediatric endocrinologist at Amsterdam University Medical Centers, the Netherlands, passed away on March 9, 2021 at the age of 54 years, six months after she was diagnosed with an untreatable form of cancer. Her untimely death is not only a great loss for her family, friends and colleagues, but also for the Dutch and international pediatric endocrine communities.

Marie-José was born in 1966 in Haarlem, the Netherlands. She graduated as MD at the Medical Faculty of Utrecht University in 1993. Her first scientific paper was the result of a research elective on four children with a combination of growth hormone, TSH and prolactin deficiency from two families, who later were shown to be the first cases carrying pathogenic variants in *POU1F1*. She was trained in pediatrics at the Willem-Alexander Children’s Hospital of the Leiden University Medical Center (LUMC) and at the Juliana Children’s Hospital in The Hague. She started her fellowship and scientific career in pediatric endocrinology at the LUMC in 2000 under the mentorship of Dr. Wilma Oostdijk and myself. Besides a substantial number of excellent scientific papers this resulted in co-founding the Leiden Working Group on Genetics of Growth in 2006. In 2007 she successfully defended her PhD thesis “Genetic Disorders in the Growth Hormone-IGF-I Axis” at Leiden University.

In 2008, she started a new position as pediatric endocrinologist at the Department of Pediatrics, VU University Medical Center (VUmc), Amsterdam, recently joined with that of the University of Amsterdam (Amsterdam University Medical Centers), in close collaboration with Prof. Paul van Trotsenburg, Dr. Joost Rotteveel and other colleagues. Together with a geneticist she initiated a novel joint outpatient clinic for children with growth disorders. She was an excellent clinician, and was universally praised by her patients and their parents.

Marie-José made many important contributions to clinical pediatric endocrine research, in particular on genetic defects of the growth hormone-IGF-I axis associated with short or tall stature. For example, she described the first case carrying a homozygous pathogenic *IGF1* variant (1), the first case with a homozygous *STAT5B* defect without severe immunological disturbance (2), and cases with homozygous *IGFALS* variants, heterozygous *IGF1R* variants and heterozygous *IGF1* variants. She also described a novel cause of growth hormone insensitivity due to a genetic variant in the gene encoding IkappaB, and a child with tall stature caused by a duplication of *IGF1R*. Based on a large group of patients with heterozygous loss-of-function *IGF1R* variants, she reported a clinical score to select short children for genetic testing of *IGF1R* (3).

Marie-José was also actively involved in national professional organizations, a.o. by serving as secretary to the Dutch Growth Hormone Advisory Group (2010-2014). From 2014 onward, she served as principal investigator of a nation-wide study on the effect of discontinuing recombinant human growth hormone treatment in adolescents who showed a normal result of a GH stimulation test in mid-puberty, in contrast to a low GH peak in childhood. Besides her passion for clinical and scientific pediatric endocrinology, Marie-José also had a keen interest in improving education in pediatric endocrinology, nationally as well as internationally and in developing novel general training programs for pediatric residents.

**Figure:**
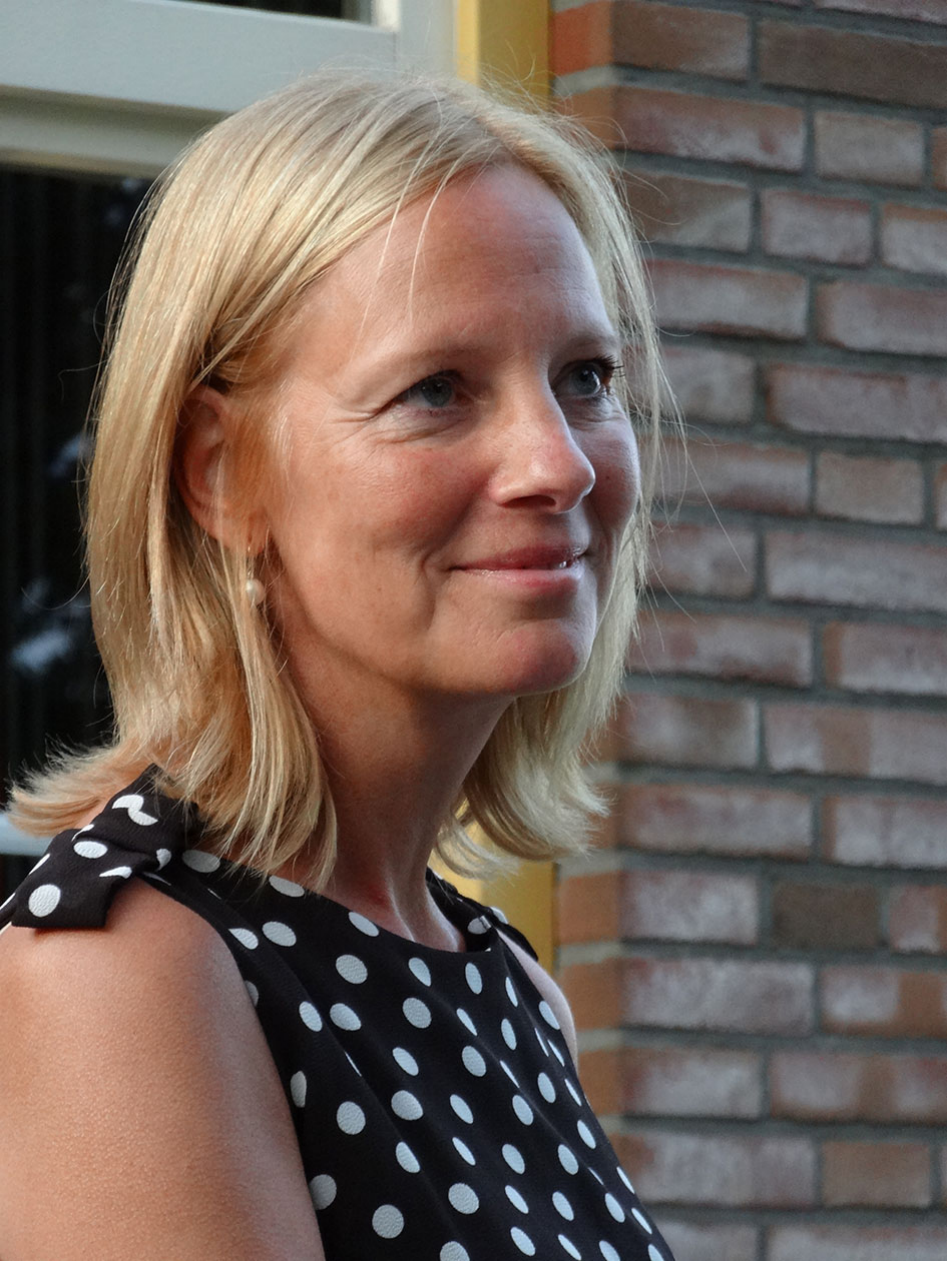
Marie-José Walenkamp at 49 years of age.

We are very sad to miss Marie-José’s kind and inspiring personality, and she is missed by many others. She was an exceptional role model for women in medicine and science.

## Author Contributions

The author confirms being the sole contributor of this work and has approved it for publication.

## Conflict of Interest

The author declares that the research was conducted in the absence of any commercial or financial relationships that could be construed as a potential conflict of interest.

## Publisher’s Note

All claims expressed in this article are solely those of the authors and do not necessarily represent those of their affiliated organizations, or those of the publisher, the editors and the reviewers. Any product that may be evaluated in this article, or claim that may be made by its manufacturer, is not guaranteed or endorsed by the publisher.
